# Cannabinoid receptor 2 agonist attenuates pain related behavior in rats with chronic alcohol/high fat diet induced pancreatitis

**DOI:** 10.1186/1744-8069-10-66

**Published:** 2014-11-17

**Authors:** Liping Zhang, Robert H Kline, Terry A McNearney, Michael P Johnson, Karin N Westlund

**Affiliations:** Department of Physiology, MS-508 College of Medicine, University of Kentucky, 40526-0298 Lexington, KY USA; Neuroscience/Pain, Lilly Research Laboratories, Eli Lilly and Company, 46285 Indianapolis, IN USA

**Keywords:** Alcohol, CB2, Pain, Ki67, Hyperalgesia, Pancreas, Behavioral testing, 44°C hotplate test, Tissue repair, High fat

## Abstract

**Background:**

Chronic Pancreatitis (CP) is a complex and multifactorial syndrome. Many contributing factors result in development of dysfunctional pain in a significant number of patients. Drugs developed to treat a variety of pain states fall short of providing effective analgesia for patients with chronic pancreatitis, often providing minimal to partial pain relief over time with significant side effects. Recently, availability of selective pharmacological tools has enabled great advances in our knowledge of the role of the cannabinoid receptors in pathophysiology. In particular, cannabinoid receptor 2 (CB2) has emerged as an attractive target for management of chronic pain, as demonstrated in several studies with inflammatory and neuropathic preclinical pain models. In this study, the analgesic efficacy of a novel, highly selective CB2 receptor agonist, LY3038404 HCl, is investigated in a chronic pancreatitis pain model, induced with an alcohol/high fat (AHF) diet.

**Results:**

Rats fed the AHF diet developed visceral pain-like behaviors detectable by week 3 and reached a maximum at week 5 that persists as long as the diet is maintained. Rats with AHF induced chronic pancreatitis were treated with LY3038404 HCl (10 mg/kg, orally, twice a day for 9 days). The treated animals demonstrated significantly alleviated pain related behaviors after 3 days of dosing, including increased paw withdrawal thresholds (PWT), prolonged abdominal withdrawal latencies (ABWL), and decreased nocifensive responses to noxious 44°C hotplate stimuli. Terminal histological analysis of pancreatic tissue sections from the AHF chronic pancreatitis animals demonstrated extensive injury, including a global pancreatic gland degeneration (cellular atrophy), vacuolization (fat deposition), and fibrosis. After the LY3038404 HCl treatment, pancreatic tissue was significantly protected from severe damage and fibrosis. LY3038404 HCl affected neither open field exploratory behaviors nor dark/light box preferences as measures of higher brain and motor functions.

**Conclusion:**

LY3038404 HCl, a potent CB2 receptor agonist, possesses tissue protective and analgesic properties without effects on higher brain function. Thus, activation of CB2 receptors is suggested as a potential therapeutic target for visceral inflammation and pain management.

## Background

Chronic Pancreatitis (CP) is a complex and multifactorial syndrome. Many contributing factors can result in abnormal pain etiology, including both peripheral and central pain processing and structural abnormalities of the pancreatic gland [1]. The higher incidence in men reported nationally is attributed not only to alcohol and tobacco abuse as risk factors, but a genetic prevalence in men to have the CLDN2 risk factor DNA variant with alcohol associated chronic pancreatitis (47%) [2–5]. Pain management in CP is challenging and often leads to time-consuming and unsatisfactory approaches to treatment with an unpredictable outcome. Despite the availability of analgesics approved for chronic or neuropathic pain, current analgesics only provide partial pain relief and/or produce significant side effects. Recently, availability of selective pharmacological tools has enabled great advance of our knowledge of the role of cannabinoid receptor 2 (CB2) in pathophysiology. The cannabinoid receptors are a class of cell membrane receptors under the G protein-coupled receptor superfamily. There are currently two known subtypes, termed CB1 and CB2. The CB1 receptors are expressed mainly in the brain (central nervous system or "CNS"), but also in the lungs, liver and kidneys. CB2 receptors are largely restricted to immune and hematopoetic cells, although functionally relevant expression has been found in specific regions of the brain and in myocardium, gut, endothelial, vascular smooth muscle and Kupffer cells, exocrine and endocrine pancreas, bone, reproductive organs/cells, and in various tumors [6–9]. Evidence suggests that there are novel cannabinoid receptors, that is, non-CB1 and non-CB2, which are expressed in endothelial cells and in the CNS [10].

Initially the interest in cannabinoid receptors as potential targets for chronic pain was limited to cannabinoid receptor 1 (CB1), with the aim of identifying an agonist suitable for drug development. Unfortunately analgesia induced by CB1 agonists is associated with undesirable central nervous system side effects which have hampered their progress for therapeutic development [11]. More recently, the type 2 cannabinoid receptor (CB2) has emerged as an interesting target alternative to CB1. Its expression is upregulated in response to tissue or nerve injury, and in preclinical pain models the activation of this receptor induces significant analgesia without overt side effects [12, 13].

The most likely cellular targets and executors of the CB2 receptor-mediated effects of endocannabinoids or synthetic agonists are proposed to be the immune and immune-derived cells (e.g. leukocytes, various populations of T and B lymphocytes, monocytes/macrophages, dendritic cells, mast cells, microglia in the brain, Kupffer cells in the liver, etc.). However, the number of other potential cellular targets is expanding, now including epithelial, endothelial and smooth muscle cells, fibroblasts of various origins, cardiomyocytes, and certain neuronal elements of the peripheral or central nervous systems [7, 14–16]. In the brain, CB2 receptors are predominately expressed by microglial cells, where their role remains unclear [7, 12, 14, 15, 17, 18] but is suggestive of an anti-inflammatory role. In particular, CB2 for chronic pain treatment has been successfully demonstrated by several studies with inflammatory and neuropathic preclinical pain models [19, 20]. While the CB2 receptors are involved in mediating analgesic effects in the peripheral nervous system, these receptors are not expressed by nociceptive sensory neurons [7]. At present CB2 receptors are believed to exist on an undetermined, non-neuronal cell in the vicinity of peripheral nerve terminals. Possible candidates in the pancreas include mast cells and innate immune stellate cells which are known to facilitate the inflammatory response. Cannabinoid mediated inhibition of these responses may be caused by a decrease in the receptor perception of noxious stimuli [10, 21, 22]. Recent studies showed that CB1 and CB2 are also expressed on pancreatic acinar cells, and the relatively nonselective CB1/CB2 agonist HU210 ameliorates acute experimental pancreatitis by systemic administration [23]. During acute pancreatitis, an upregulation especially of CB2 on apoptotic cells has been shown, and activation of cannabinoid receptor 2 attenuated the acute pancreatitis [24]. Little is known about the effects of CB2 that impact inflammation and pain in chronic pancreatitis.

In the present study, a novel CB2 receptor agonist LY3038404 HCl was evaluated for therapeutic efficacy in the AHF chronic pancreatitis rat model. The major finding of the present study is that the potent CB2 receptor agonist LY3038404 HCl possesses tissue protection and analgesic properties. No side effects on higher brain function were observed. Thus, activation of CB2 receptors is suggested as a potential therapeutic target for visceral inflammation and pain management.

## Results and discussion

### AHF diet induced chronic pancreatitis histopathology

The AHF pancreatitis rats gain less body weight per week compared to control rats fed standard rodent chow. The final weight gain plateau in AHF fed rats was significantly less than that of control rats (data not shown), but was within the 20% safety limit of the American Veterinary Medical Association Guidelines on Euthanasia (2013) and local institutional Animal Care and Use Committee (IACUC) guidelines. In patients, chronic pancreatitis can be accompanied by weight loss due to symptoms (abdominal pain, nausea and anorexia), decreased pancreatic enzymes and nutrient absorption, and alcoholic dietary deficiencies [25].

### Chronic pancreatitis pathology with fibrosis and vacuolization in AHF fed rats

**Figure 1:**
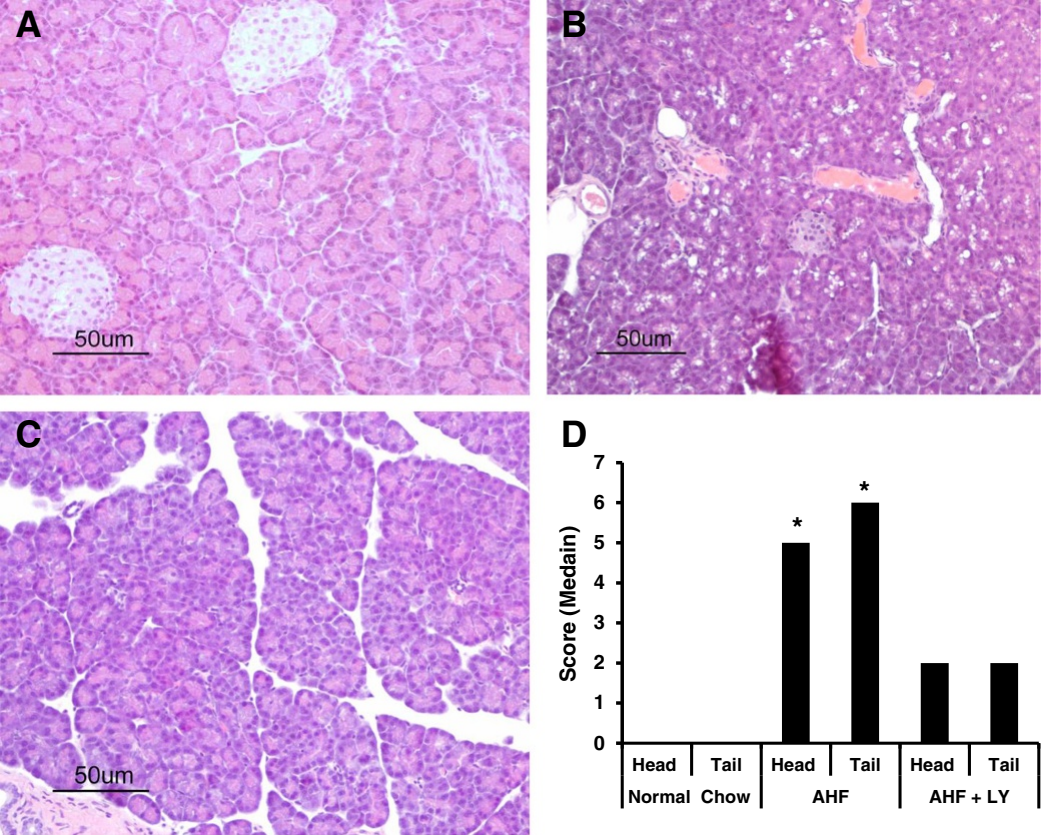
**AHF diet induced chronic pancreatitis pathology and protection by LY3038404 HCl.** Representative bright field images of pancreatic paraffin sections stained with H & E. **A**. Normal chow fed rats had normal pancreatic architecture with evenly distributed acini and islets of Langerhans. **B**. Rats with AHF diet chronic pancreatitis had abnormal pancreatic tissue architecture including global degeneration (with blurred cell borders) and multiple vacuolization sites due to fat deposition. **C**. LY3038404 HCl treated rats with AHF pancreatitis showed improvement of tissue damage. **D**. The histogram shows the histological severity rating scores (HHS) of different treatment groups. The score is a total of the different parameters for each animal averaged within each group, expressed as a median (One-way ANOVA, Kruskal-Wallis, *p < 0.05).

**Figure 2:**
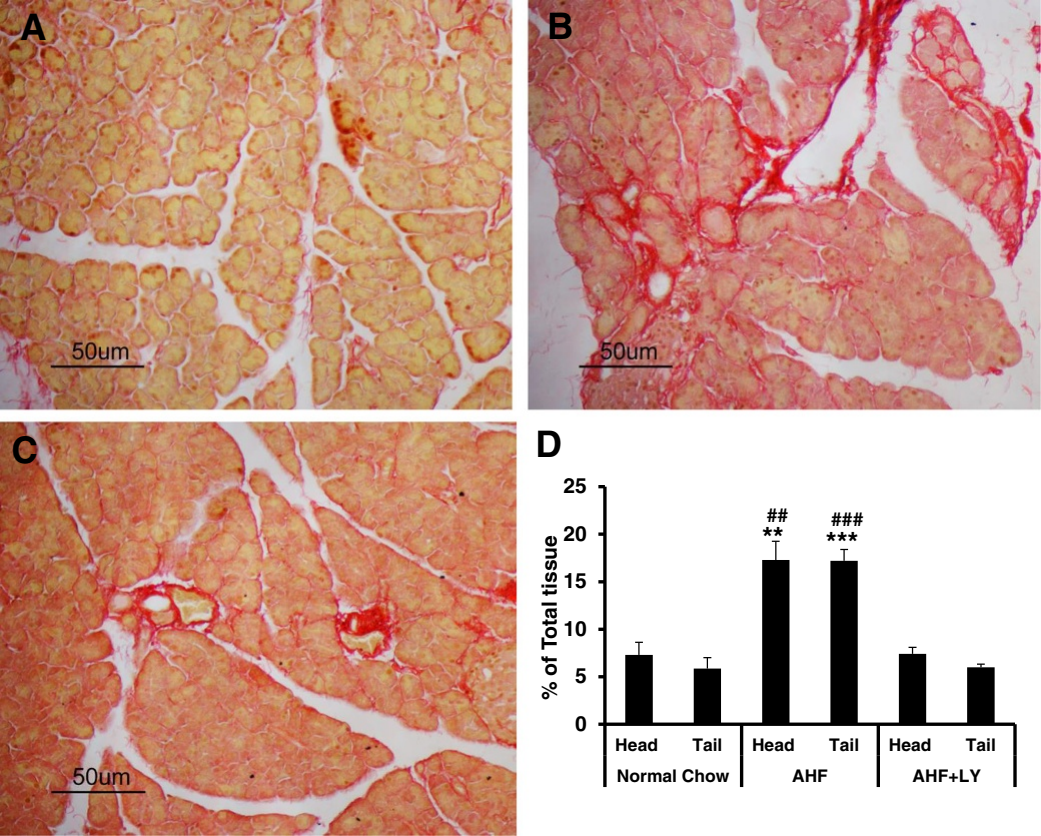
**Chronic pancreatitis pathology with fibrosis and LY3038404 HCl effectively reduced pancreatic tissue fibrosis.** Representative bright field images of Sirius red stained pancreatic sections. **A**. Normal chow control group had little fibrosis. **B**. AHF pancreatitis group rats had an invasive fibrous network around the acini, blood vessels, and ducts. **C**. LY3038404 HCl treated rats with AHF pancreatitis showed an improvement with much less tissue fibrosis. **D**. The histogram shows the statistically significant increase in percent fibrosis relative to total tissue area in AHF fed rats with chronic pancreatitis compared to the controls (n = 6/group, **p < 0.01 and ***p < 0.001). LY3038404 HCl treatment effectively protected rats with AHF pancreatitis from severe pancreatic tissue damage (n = 6/group, ^##^ *p* < 0.01 and ^###^ *p* < 0.001).

Pancreas tissues were taken from both normal chow control and AHF pancreatitis rats in week 12 at the end of the experiment. Pancreas from normal chow fed rats had normal architecture with evenly distributed acini and islets of Langerhans (Figures 1A; 2A). Histological assessment of pancreas from rat with AHF pancreatitis revealed global gland degeneration (acinar and islet cell atrophy), abundant vacuolization (fat deposition) (Figure 1B), as well as invasive periductal, interlobular, and intralobular fibrosis (Figure 2B). Inflammatory cells were not detected in the pancreas tissue sections.

The total overall histology severity score (HSS) for the pancreas of the AHF fed group was 5±0 (median = 5) (n = 6) for the head (duodenal lobe) and 6.33±0.33 (median = 6) (n = 6) for the tail (splenic lobe) (Figure 1D). This was significantly different compared to an HSS of 0 (median = 0) for the normal chow control group (n = 6, *p* < 0.05, One-way ANOVA, Kruskal-Wallis).

Quantitative analysis of the percentage of the total pancreatic area positive for collagen staining (reflecting fibrosis) demonstrated a significant increase of >17% in AHF fed rats, compared to <7% in the control group (Figure 2D). The total fibrosis in the pancreas head was 17.29±1.9% and in the tail was 17.20±1.2% from the AHF pancreatitis rats. This was a statistically significant increase compared to the controls (n = 6, *p* < 0.01and *p* < 0.001, respectively, Student’s t-test).

These data show that the AHF induced chronic pancreatitis rat model featured a globally disrupted pancreatic pathology; including acinar and islet cell atrophy, progressive accumulation of lipid droplets in acinar cells (vacuolization), and periductal, interlobular and intralobular fibrosis. Pancreatic infiltration by immunocompetent inflammatory cells was not detected in any of the tissue sections. These chronic morphological changes in rats with AHF chronic pancreatitis are consistent with pathological changes described in clinical samples from patients with alcoholic pancreatitis [26, 27].

#### LY3038404 HCl preserved pancreatic architecture in rats with AHF pancreatitis

LY3038404 HCl effectively blocked the widespread progress of pancreatic tissue degeneration described above for chronic AHF pancreatitis rats (Figure 1C and D). The HSS was 2±0 (median = 2) for the head and 2.33±0.33 (median = 2) for the tail in the AHF + LY3038404 HCl treated group. This was not different from the normal chow control group and was significantly improved compared to the HSS of 5 – 6 range in AHF pancreatitis rats without drug treatment group (p < 0.05, One-way ANOVA, Kruskal-Wallis).

The total collagen staining area was significantly decreased to 7.4±0.68% in the head and 5.98±0.33% in the tail of the AHF + LY3038404 HCl treated group compared to the untreated AHF rats with pancreatitis (Figure 2C and D) (*p* < 0.01 and *p* < 0.001, One-way ANOVA, Tukey's Multiple Comparison test). This was within range of the percentage obtained from the control rats. Thus, the decreased percentage of the total pancreatic area staining positively for collagen and the overall pancreatic architecture demonstrated improvement in the LY3038404 HCl treated rats.

#### Increased Ki67 cell proliferation protein expression in injured pancreas

**Figure 3:**
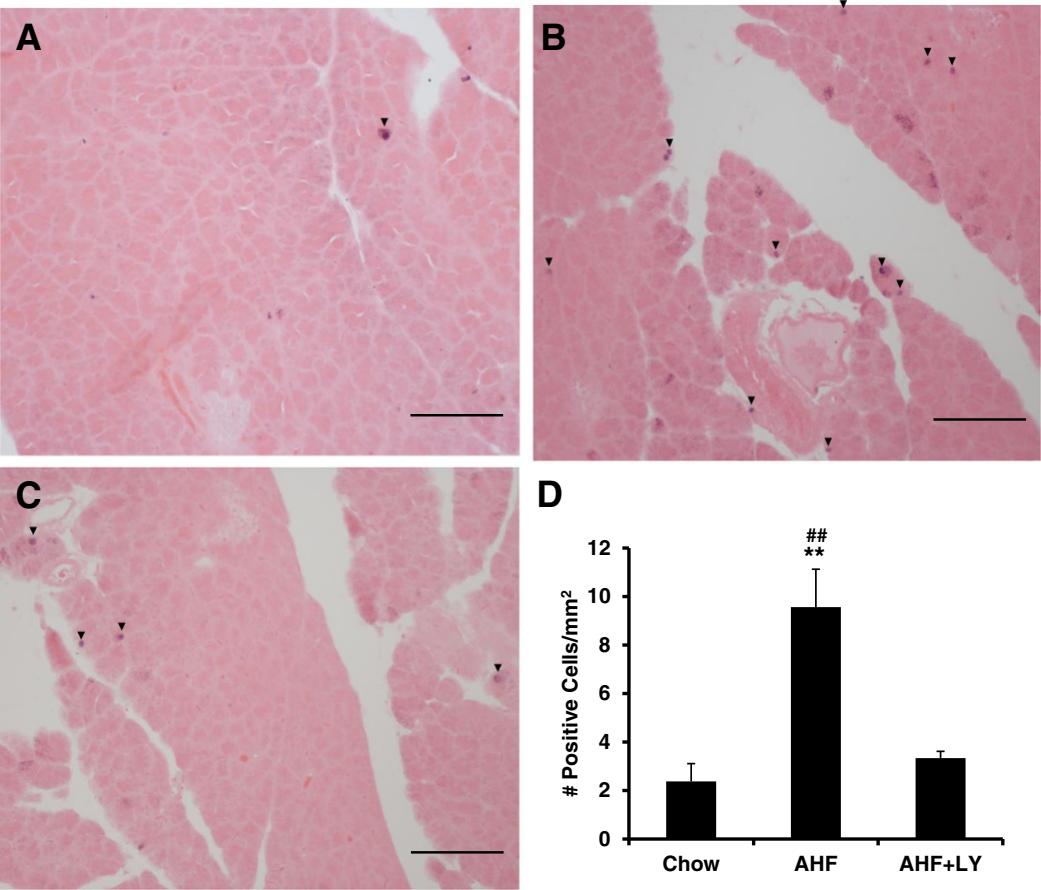
**Increased Ki67 immunoreactivity in pancreatic tissue and LY3038404 HCl effectively inhibited the expression of Ki67.** Pancreas tissue sections are shown. **A**. Control pancreas tissue has low basal Ki67 expression in the acinar cell nuclei. **B**. Numerous Ki67 positive cell nuclei are evident in the pancreas of rat with AHF pancreatitis. **C**. Chronic treatment with LY3038404 HCl effectively blocked Ki67 expression. **D**. The histogram shows the cell count per mm^2^ pancreas tissue section. There is a statistically significant difference in Ki67 staining between normal chow fed animals and animals with AHF pancreatitis (One-way ANOVA, Tukey's Multiple Comparison test, n = 6/group, **p < 0.01). LY3038404 HCl treatment effectively reduced Ki67 expression in rats with AHF pancreatitis (n = 6/group, ^##^ *p* < 0.01). Scale bar = 50 μm.

There were few basal Ki67-positive cells observed in pancreas of normal chow fed control rat (2.37 ± 0.7/mm^2^) (Figure 3A). The cell proliferation protein Ki67 was evident in the AHF fed animals mainly expressed in the nuclei of acinar cells and in some periductal epithelial cells (Figure 3B). The increased number of Ki67 positive cells in the injured pancreas of AHF fed rats was statistically significant compared to control chow fed rats (9.56 ± 1.6/mm^2^) (Figure 3D) (*p* < 0.01, One-way ANOVA, Tukey's Multiple Comparison test, n = 6/group). This was consistent with other reports of tissue injury and repair activities in acute as well as in chronic pancreatitis following long term insult to the pancreas [28–30]. The increased presence of the proliferation biomarker Ki67 is suggestive of cellular events that favor progression to tumor development [31].

#### LY3038404 HCl diminishes Ki67 indicated cell proliferation

Pancreatic immunostaining for Ki67 positive nuclei revealed a similar mean number of positive nuclei comparing LY3038404 HCl treated pancreatic group with the normal control rats (Figure 3A, C and D) (3.34 ± 0.27/mm^2^ vs 2.37 ± 0.74/mm^2^; *p* > 0.01, One-way ANOVA, Tukey's Comparison test).

Thus, orally administered LY3038404 HCl exerted protective effects in the pancreas, significantly reducing Ki67 upregulation and AHF induced tissue damage. The CB2 agonist treatment given in week 6 after the start of AHF diet significantly protected the pancreatic tissues from severe degeneration, vacuole formation and fibrosis.

LY3038404 HCl’s tissue protective effect is most likely related to key roles of CB2 receptor activators as antioxidants and inhibitors of cell proliferation shown by others [32]. This tissue protective effect is supported by the preponderance of studies to date indicating that the CB2 cannabinoid receptor is linked to cytochrome P450 mono-oxygenases [33] and is a modulator of the majority of immune functional responses [12, 14, 34]. Michler et al. [24] demonstrated the importance of CB2 activation in protective modulation involving MAPK p38 and MK2 dependent pathways in an acute pancreatitis model. A similar study also demonstrated that local peripheral CB2 receptor activation inhibits inflammation and inflammatory hyperalgesia [35]. AM1241, a selective agonist of CB2, fully reversed carrageenan-induced inflammatory heat hyperalgesia when injected directly into an inflamed paw. In contrast, AM1241 injected into the contralateral paw had no effect, demonstrating CB2 receptor activator has a local peripheral effect.

### Pain related behaviors induced in rats with AHF diet chronic pancreatitis

#### Secondary hindpaw mechanical allodynia

**Figure 4:**
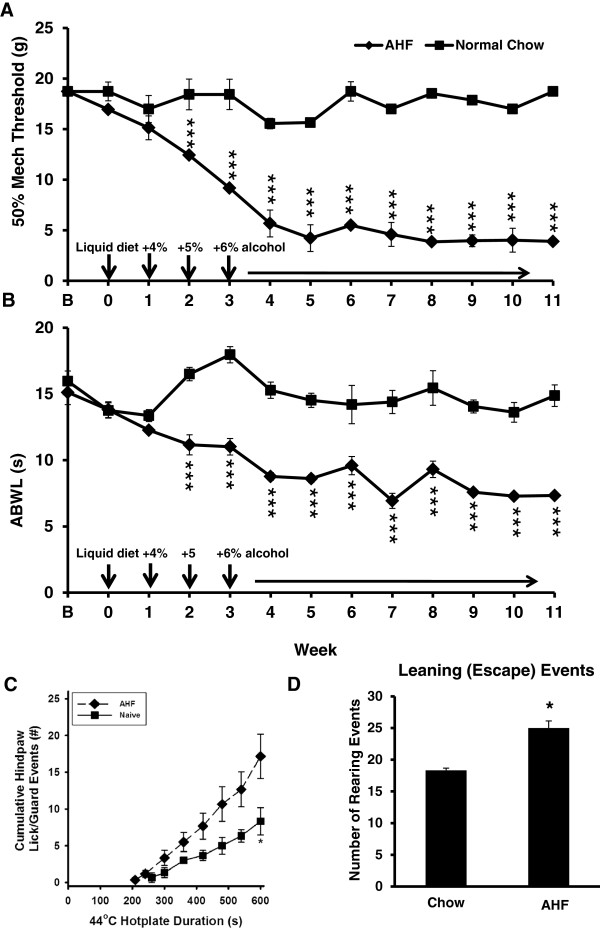
**Rats with AHF chronic pancreatitis developed secondary hypersensitivity. A. Hindpaw Mechanical Allodynia.** The baseline for the 50% paw withdrawal threshold to mechanical stimulation (von Frey fiber) was similar in control chow fed rats and the AHF fed group. The mechanical threshold was progressively decreased in the AHF fed group starting from week 3 and reaching maximal hypersensitivity at week 5. The paw mechanical allodynia remained through the entire experiment time course (Two-way ANOVA, Bonferroni post-test,***p < 0.001). **B**. Abdominal Secondary Heat Hyperalgesia. The baseline for abdominal withdrawal latency (ABWL) was similar between control chow and AHF fed groups. Secondary heat hyperalgesia developed on the abdominal skin area in the fourth week of AHF feeding. The abdominal withdrawal latency (ABWL) baseline of 13.39±1.4 s was decreased to 8.77±0.28 s and remained at this level throughout the experimental time course. This was a statistically significant difference compared to the control chow fed group (Two-way ANOVA, Bonferroni post-test, ***p < 0.001). **C**. and **D**. 44°C Hotplate Hypersensitivity Assay. **C**. The nocifensive responses were recorded for 10 min (1 minute per bin). The event/time curve in rats with pancreatitis showed a statistically significant left-ward shift compared to that of the normal chow fed control group (Two-way ANOVA, Bonferroni post-test, n = 6/group, *p < 0.05). **D**. The rearing events on the hotplate were significantly increased for rats with AHF pancreatitis compared to normal chow fed rats (t-test, *p < 0.05).

The baseline for the 50% paw withdrawal threshold to mechanical stimulation (von Frey fiber test) was initially similar for both the control chow fed and the AHF fed rat groups, i.e. 18.57±0.09 g for both feet. The AHF fed rats developed hindpaw mechanical hypersensitivity three weeks after the start of the AHF diet. The mechanical threshold was progressively decreased from week 3, reached maximum hypersensitivity at week 5, and remained low throughout the subsequent weeks with continuous feeding of the AFH diet. The paw withdrawal threshold was decreased to 4.21±0.39 g bilaterally, a significant difference compared to that of the control chow fed group (17.10±0.09 g) (*p* < 0.001, Two-way ANOVA, Bonferroni post-test) (Figure 4A).

#### Secondary abdominal heat hyperalgesia

The baseline abdominal withdrawal latencies (ABWL) of the control chow and AHF fed groups were similar (control chow (n = 6) 13.6 ±1.19 s vs. AHF (n = 12) 13.39±1.4 s). The secondary heat hyperalgesia on the abdominal skin developed in the fourth week in AHF diet fed rats. The ABWL decreased from baseline 13.39±1.4 s to 8.77±0.28 s, compared to the unchanged ABWL in the control chow fed group (13.41±1.2 s) (*p* < 0.001, Two-way ANOVA, Bonferroni post-test). The ABWL persisted at this low level for the remainder of the experimental time course (Figure 4B).

#### Increased nocifensive responses to noxious 44°C stimulation

Nocifensive responses to the modified 44°C hotplate test were recorded for all groups. To normalize the skin temperature of the feet at testing, all rats were first placed on a 38°C hotplate for 10 min, then immediately transferred onto the 44°C hotplate. The nocifensive responses recorded were as follows (1 minute/bin): hindpaw licking/guarding events; the first response latency (hind-paw withdrawal latency) and rearing events. The hindpaw withdrawal response latency to the noxious heat stimuli (44°C) in control rats was 260.53±27.2 s. The accumulated hindpaw lick/guarding events occurred an average of 8.33±1.9/10 min. In contrast, the first hindpaw withdrawal latency in rats with AHF pancreatitis started as early as 208.6±30.2 s. The average of accumulated hindpaw licking/guarding events was 17.2±3/10 min. The event/time curve clearly showed a significant left-ward shift compared to that of the control chow group (*p* < 0.05, Two-way ANOVA, Bonferroni post-test, n = 6/group) (Figure 4C). The rearing or wall leaning posture was recorded during the 10-minute 44°C hotplate assay. Rats with AHF pancreatitis displayed more rearing events (25 ± 1.3/10 min) than control chow fed rats (18.33 ± 0.6/10 min) (Figure 4D; *p* < 0.05, Student’s t-test). This assay documented the development of secondary heat hyperalgesia and increased escape responses in rats with AHF pancreatitis.

Thus, in this study, rats with AHF induced chronic pancreatitis demonstrated progressively increasing visceral pain-like behaviors, as well as secondary hypersensitivity. Secondary hypersensitivity was indicated by decreased mechanical threshold (secondary mechanical allodynia) and shortened response latency to noxious heat stimuli (secondary heat hyperalgesia), tested in two somatic referred pain regions (paw and abdomen). Rats with chronic pancreatitis displayed more complex pain-like behaviors and escape activity in the 44°C hotplate assay. These behaviors included frequent hindpaw lifting and licking (secondary heat hyperalgesia) and increased rearing events (attempted escape) in response to the noxious heat stimuli.

### LY3038404 HCl effectively attenuated pain related behaviors

Long term oral feeding of LY3038404 HCl effectively attenuated pain related behaviors in rats with AHF pancreatitis.

#### Paw Withdrawal Threshold (PWT)

**Figure 5:**
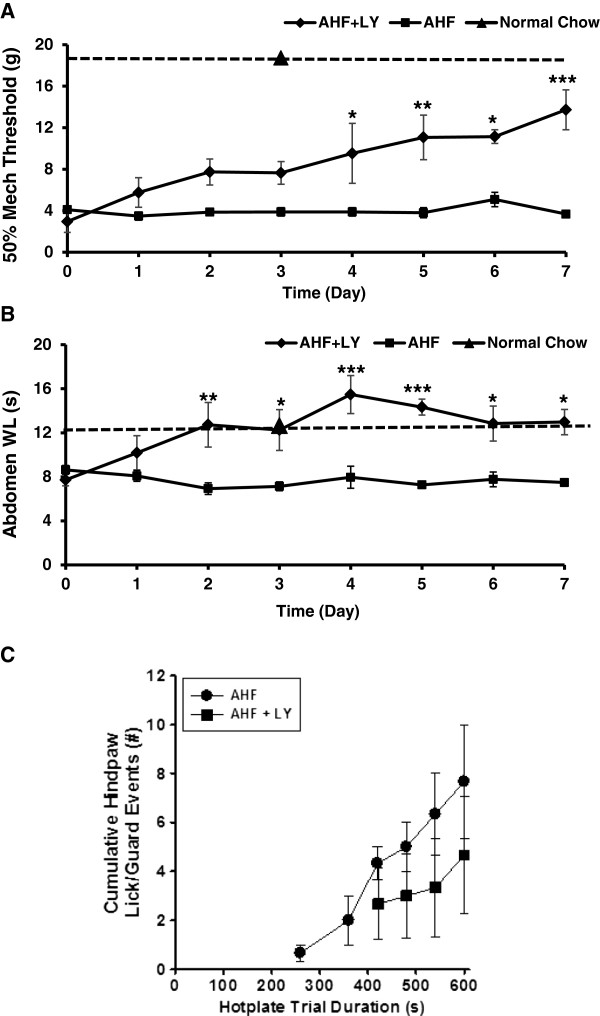
**LY3038404 HCl attenuated evoked pain related behaviors.** LY3038404 HCl (10 mg/kg, oral, twice a day, 7 days), effectively attenuated pain related behaviors. **A**. Secondary paw mechanical allodynia was reduced (AHF vs. AHF + LY3038404 HCl, n = 6/group, Two-way ANOVA, Bonferroni post-test, *p < 0.05; **p < 0.01; ***p < 0.001). **B**. Secondary abdominal heat hyperalgesia was reduced (AHF vs. AHF + LY3038404 HCl, Two-way ANOVA, Bonferroni post-test, n = 6/group *p < 0.05, **p < 0.01, ***p < 0.001). **C**. LY3038404 HCl (10 mg/kg, twice daily, 8 days) reduced hypersensitivity to noxious 44°C hindpaw stimulation (10 min). LY3038404 HCl prolonged the first response latency and reduced the cumulative amount of hind-paw lick or guard reflex events. Compared to rats without drug treatment, the event and time curve of drug treatment group showed a right-ward shift.

LY3038404 HCl was given twice a day for 7 days (10 mg/kg, p.o.). Pain related behavioral testing (PWT and ABWL) was performed one hour after the second dosing of the day and similar testing was repeated every day for the entire drug treatment course (7 days). Progressive elevations of paw mechanical thresholds were noted in the AHF pancreatitis rats after three days of receiving LY3038404 HCl treatment. The 50% PWT (in grams force) on both feet was increased to 14.11±2.3 g in the group with drug treatment on the seventh day. This was a statistically significant increase from the non-treatment AHF pancreatitis group (4.69±0.39 g) (*p* < 0.001, n = 6/group, Two-way ANOVA, Bonferroni post-test) (Figure 5A).

#### Abdominal Withdrawal Latency (ABWL)

ABWL was assessed immediately after the PWT testing each day. The abdominal withdrawal latency was progressively prolonged 3 days after LY3038404 HCl treatment. The average ABWL before drug treatment was 7.05±0.5 s and increased to 11.041±0.59 s at the end of the 7 day drug treatment. There was a statistically significant difference in the ABWL of AHF pancreatitis rats between the LY3038404 HCl treatment group (11.041 ± 0.59 s) and the rats without drug treatment (7.18±0.59 s) (*p* < 0.05, n = 6/group, Two-way ANOVA, Bonferroni post-test) (Figure 5B).

#### Nocifensive responses to noxious heat stimuli (44°C hotplate)

On treatment day 8, LY3038404 HCl significantly prolonged the first response latency (421±90 s) and reduced the cumulative amount of hind-paw licking or guarding reflex events (4.66±2.4/10 min) compared to AHF pancreatitis rats without drug treatment (259.6±60 s; 7.66±2.3/10 min). The event/time curve of the drug treatment group showed a right-ward shift compared to the nondrug treatment group (Figure 5C).

### Rats with AHF pancreatitis display decreased anxiety-like behaviors in two compartment light/dark box testing

It is known that Fischer 344 strain rats innately exhibit greater anxiety and fear-motivated behaviors (e.g. relatively low open field exploratory behaviors, social contact, and low running wheel activity) compared to many other rat strains [36, 37]. We used the light–dark box test to assess anxiety-like phenotypes. Rats chronically fed the AHF diet and control rats fed normal chow were placed into the light–dark box and monitored for 10 min.

**Figure 6:**
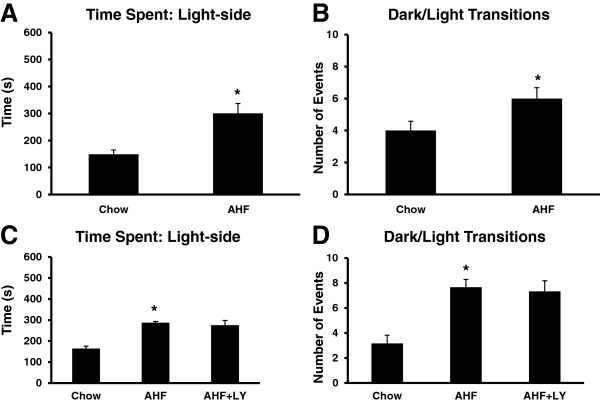
**LY3038404 HCl did not affect light/dark box preference. A**. Rats with AHF pancreatitis spent more time in the light compartment (300.77±36.45 s) than did rats fed regular chow (148.97±15.7 s) (t-test, *p < 0.05). **B**. The number of transitions between the light and dark compartments was increased in rats with AHF pancreatitis (6±0.66/10 min) compared to normal chow controls (4±0.57/10 min, t-test, *p < 0.05). **C**. LY3038404 HCl (10 mg/kg, oral feeding, twice daily, for 9 days) did not affect the light/dark preference behaviors of AHF pancreatitis rats. The time spent in the light compartment for rats with AHF pancreatitis without drug treatment (287.1±6 s) was similar to that of the drug treatment group (275.4±22.3 s) (One-way ANOVA, *p < 0.05). **D**. The number of transitions between the light and dark compartments for rats with AHF pancreatitis (7.33±0.85/10 min) was significantly greater than control chow fed rats (3.2 ± 0.6/10 min, *p < 0.05), but there was no effect with drug treatment (7.67±0.61/10 min) (Chow vs. AHF, AHF vs. AHF + LY3038404 HCl, One-way ANOVA, Tukey's Multiple Comparison test, n = 6/group, *p* > 0.05).

During the assessment in the test apparatus, the control chow fed rats preferred the dark. They remained in the dark chamber for 70% of the total testing time as previously reported by others [38]. However, AHF pancreatitis rats displayed 50/50 light/dark occupancy and had more exploratory activity transitions between the light and dark chambers. The AHF fed rats with pancreatitis spent a significantly increased amount of time in the light compartment compared to the control rats fed chow (300.77±36.45 s vs. 148.97±15.7 s) (*p* < 0.05, Student’s t-test) (Figure 6A). In rats with AHF pancreatitis, the greater number of accompanying transitions between the light and dark compartments (6 ± 0.66/10 min) was also statistically significant compared to control chow fed rats (4±0.57/10 min) (p < 0.05, Two-tailed t-test) (Figure 6B).

This behavior in the AHF rats with pancreatitis may be due to masking of any test related anxiety-like behaviors as a result of the long term ethanol consumption [38–42]. McCool et al. [42] showed that chronic ethanol exposure increased the functional expression of GABAA receptors in the neurons of basolateral amygdala which is included in the brain alcohol reward system. The facilitation of GABAA receptors during chronic ethanol exposure might help explain the maintenance of ethanol’s anti-anxiety effects during chronic ethanol exposure.

### LY3038404 HCl did not affect higher brain functions

**Figure 7:**
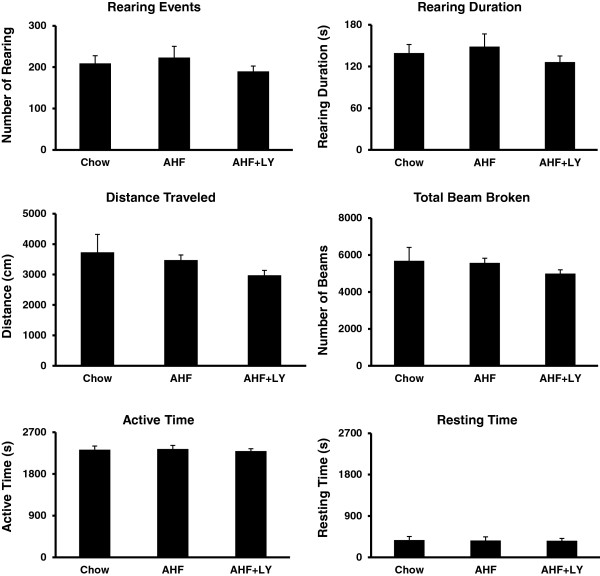
**LY3038404 HCl did not affect rat exploratory activities.** LY3038404 HCl (10 mg/kg, twice daily, 9 days) did not affect any parameters of the rat open field exploratory behavioral activities (rearing events, rearing duration, total activities, distance traveled, total active time and total resting time (n = 6/group). There were no significant differences in the responses with or without drug treatment (*p* > 0.05).

Long term oral feeding of LY3038404 HCl had little or no effect on light dark preference (Figure 6 C&D) and open field exploratory (Figure 7) behaviors in rats with AHF pancreatitis.

#### Two chamber dark/light box testing

During a 10 min time course exposure to the two chamber light/dark preference test apparatus, rats fed the control chow allocated the majority of their time to the dark chamber with only 164.03±11.23 s occupancy in the light chamber (Figure 6C & D) (*p* < 0.05, One-way ANOVA, Tukey's Multiple Comparison test). Rats with AHF pancreatitis showed reduced anxiety-like behaviors indicated by almost 50/50 occupancy of both the light and the dark chambers, with a light chamber preference of 287.1±6.09 s occupancy. AHF pancreatitis rats had an increased number of light/dark chamber transitions (7.67±0.61/10 min) compared to control chow fed rats (3.2±0.6/10 min).

The LY3038404 HCl (10 mg/kg, twice daily, for 9 days) treatment had no effect in AHF pancreatitis rats either for light/dark preference or the number of transitions between chambers, i. e. time spent in light: 275.4±22.3 s, the number of transition: 7.33±0.85/10 min (*p* > 0.05, AHF vs. AHF + LY3038404 HCl, One-way ANOVA) (n = 6/group) (Figure 6C & D).

#### Open field exploratory activities

An open field test was performed after two additional consecutive days of LY3038404 HCl treatment, to assess CB2 agonist effects on the six exploratory activities and anxiety-like behavioral measures in the rats (Figure 7). LY3038404 HCl had no significant effect on any of these parameters used as comparative assessments of higher order exploratory behavior and motor function. The following six parameters reflecting animal exploratory and motor activities were assessed: rearing events and rearing duration; total activities (by beams broken), total distance traveled and total times while active and at rest (n = 6). No significant difference in behaviors of the AHF pancreatitis group and the chow fed group (*p* > 0.05).

### Correlation analysis of histopathology and pain related behaviors

**Figure 8:**
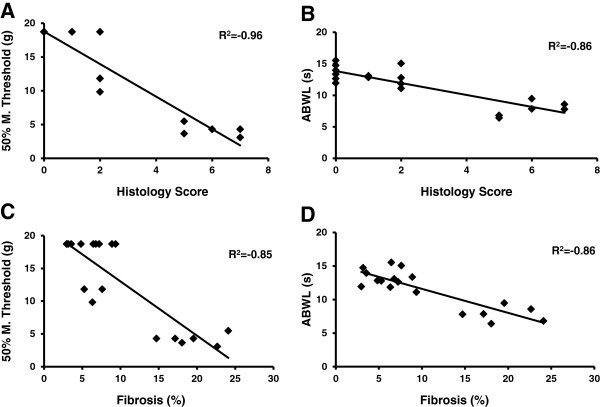
**Correlation analysis among pain behaviors and tissue pathology.** Pancreatic tissue damage and pain related behavioral measures were correlated in the three groups of rats (control chow fed rat, rat with AHF pancreatitis and rat with AHF pancreatitis + LY3038404 HCl). **A**. Animals with higher pathology scores (HSS) had lower mechanical thresholds. **B**. Animals with higher pathology scores (HSS) had shortened abdominal heat withdrawal latencies. **C**. Animals with a higher percentage of fibrosis had lower mechanical thresholds. **D**. Animals with a higher percentage of fibrosis had shortened abdominal heat withdrawal latencies.

Further analysis of the correlation coefficient (Microsoft Excel) between severity of pancreatic tissue damage (HSS and percent tissue fibrosis) and pain related nocifensive responses (mechanical hind paw withdrawal threshold and heat abdominal withdrawal latency) revealed a strong negative correlation for these two assessments. Pancreatic tissue damage severity was correlated with pain related behavioral measures for all three rat groups, control chow fed, AHF fed, and AHF + LY3038404 HCl ) (Figure 8). The AHF pancreatitis animals with higher HHS had lower mechanical threshold (Figure 8A) and a shortened heat withdrawal latency (Figure 8B). These results were also reflected when assessing collagen staining. Animals with a higher percentage of fibrosis had lower mechanical thresholds (Figure 8C) and shortened heat withdrawal latencies (Figure 8D). These correlative analyses are supportive of LY3038404 HCl as a protective treatment for reducing severe pancreatic tissue damage and hypersensitivity.

Based on the histopathology and behavioral test correlations, it is suggested that suppression of referred mechanical allodynia and heat hyperalgesia by LY3038404 HCl may be the result of the combined effects of reduced pancreatic pathology and inhibition of nociceptive peripheral nerve activity due to the long term CB2 receptor activation.

In summary, the LY3038404 HCl attenuated evoked mechanical and heat pain related behaviors, but had no effect on anxiety-like behavior. The LY3038404 HCl increased paw withdrawal threshold (PWT), prolonged abdominal withdrawal latency (ABWL), and decreased nocifensive responses to noxious 44°C hotplate stimuli. These findings are consistent with a previous study in which HU210, a synthetic agonist at CB1 and CB2, abolished abdominal pain associated with acute pancreatitis, reduced inflammation, and decreased tissue pathology in mice without producing adverse central effects [23]. A recent study demonstrated that activation of CB2 receptor inhibits bladder inflammation and associated referred mechanical hyperalgesia in a mouse cystitis model [43]. Orally administered PF-03550096 (10 mg/kg), a potent CB2 agonist, also inhibited the 2,4,6-trinitrobenzene sulfonic acid (TNBS)-induced decrease in pain threshold in a rat model of visceral (colonic) hypersensitivity [44]. These reports and the data presented here indicate that cannabinoids are generally beneficial in visceral inflammatory disorders and visceral pain.

Our data revealed that LY3038404 HCl altered neither a light/dark preference behavior nor open field exploratory activity in the AHF pancreatitis rats. This indicates LY3038404 HCl did not affect higher brain mediated behaviors and was not associated with central side effects reported for CB1 agonists in previous studies [12–15]. These findings support beneficial peripheral CB2 receptor signaling mechanisms of action for LY3038404 HCl. Activation of CB2 receptors located on peripheral terminals of nociceptors or local cells could diminish nociceptor excitability and thus block pain signaling [10, 21].

## Conclusion

The major finding of the present study is that LY3038404 HCl, a potent CB2 receptor agonist, possesses tissue protective and analgesic properties. No effects on higher brain functions were observed including the diminished fear responses induced by the alcohol diet. Thus, activation of CB2 receptors is suggested as a potential therapeutic target for pancreas protection and pain management.

## Methods

The studies were performed in accordance with the *Guide for the Care and Use of Laboratory Animals* published by the National Institutes of Health and were approved by the University of Kentucky Institutional Animal Care and Use Committee.

### Experimental design

These studies were performed in order to evaluate the therapeutic efficiency of a novel CB2 receptor agonist LY3038404 HCl in the AHF chronic pancreatitis rat model. After the nocifensive behaviors were established, i.e. six weeks after the start of the alcohol/high fat liquid diet feeding, the LY3038404 HCl was given twice a day for 7 days (10 mg/kg, p.o.). The pain related behavioral tests were performed one hour after the second drug dosing each day. Higher order preference tests were performed on two additional days of treatment. At the conclusion of the study, animal pancreatic tissues were harvested to evaluate the severity of the pancreatitis. The therapeutic efficiencies of LY3038404 HCl were evaluated by analyzing 1) pancreatic tissue HSS and fibrosis for tissue protection; 2) evoked, place preference, and open field behavioral data for determination of nociceptive control and side effects.

### Induction of chronic pancreatitis

Male Fischer 344 rats weighing between 240 – 250 g (Harlan Sprague–Dawley, Indiana) were used for this study. Rats were randomly divided into two groups: alcohol/high fat liquid diet (AHF) fed - (n = 12) and control chow fed- group (n = 6). Animals were single caged and kept in a temperature constant (23° ± 2°C) room on a 12/12 hour reversed dark–light cycle. Chronic pancreatitis was induced in animals fed an alcohol/high fat liquid diet (AHF) made from micro-stabilized rodent alcohol liquid diet mix (LD 101A; Test-Diet, Richmond, IN), composed of 3.9% fat from corn and safflower oil, 30.3% protein, 5% fiber, vitamins and minerals added as a dry powder to water and alcohol (w/v, 95% ethyl alcohol). The dose of alcohol was progressively increased from 4% to 6% as follows: 4% alcohol for the first week, 5% for second week, and 6% for the third to eighth week. Lard (8 g/per rat/per day) was added starting from the 4% alcohol week. Rats were allowed free access to water. Each rat consumed between 50–70 grams of liquid diet with alcohol per day for 12 weeks. Animals also received a daily lard supplement (8 g/day/per rat) bringing the total fat content of their diet to ~20% total daily dietary fat consumption. Control group was fed standard rodent chow (Teklab 8626, Harlan, Indiana) and they had access to food and water ad libitum. Animals were observed closely and no evidence of alcohol intoxication (no ataxia or lethargy) was noted. Body weight was monitored weekly and food consumption was monitored daily.

### Drug administration

LY3038404 HCl and dosages were provided by Eli Lilly and Company, Indianapolis IN. LY3038404 HCl powder was freshly mixed with control rat chow powder and drinking water to form a small drug pellet (≈1 g/each) one day before use. The small pellets were dried in a 50°C-oven for 2 hours.

The LY3038404 HCl treatment started after the 6th week when pain related behavior testing detected a maximum hypersensitivity in the AHF fed animals. Rats were fed LY3038404 HCl (10 mg/kg) orally twice a day for 9 consecutive days. On the testing day one pellet was given at 8:30 - 9 am, and one at 12 - 1 pm. The evoked mechanical and heat tests were performed on Days 1–7, one hour after the second dosing of the day. Place preference and open field tests were performed on Days 8 and 9, respectively.

### Histopathological assessment of pancreatitis

At the end of study, rats were anesthetized with pentobarbital (100 mg/kg, i.p.) and perfused transcardially with warm heparinized saline followed by 4% ice-cold paraformaldehyde in 0.1 M phosphate buffer solution (pH 7.4). The pancreas was post-fixed in the buffered paraformaldehyde for 48 hours, carefully dissected into 2 parts: duodenal (pancreatic head) and splenic lobes (pancreatic tail) were put into 70% alcohol. After dehydration through graded ethanol (95, 100%), the pancreas was embedded in paraffin. Pancreatic tissue sections were cut at 5 μm thickness, mounted onto gelatin coated glass slides (Super Frost Plus, VWR, Radnor, PA), and stored at room temperature for histological staining. Analysis was performed blindly on serial sections from each rat.

#### Hematoxylin and Eosin (H&E) staining

Slides were de-paraffinized with Citrosolv (Fisher, Pittsburgh, PA), hydrated with graded ethanol, rinsed in tap water, immersed in 0.1% hematoxylin (Fisher) for 1–3 min, washed in tap water for 1 min, dehydrated through in graded ethanol (50%, 70%), immersed in 0.1% eosin (Fisher) for 1 min, and then dehydrated in 90% ethanol. Finally, sections were dehydrated in 100% ethanol and coverslipped with Permount (Fisher, Pittsburgh, PA).

#### Sirius Red staining for collagen

De-waxed and hydrated paraffin sections were stained with haematoxylin for 8 minutes and then washed for 10 minutes in running tap water. Sections were stained in picro-sirius red for one hour. After two washes in acidified water, sections were dehydrated in three changes of 100% ethanol, cleared in Citrosolv (Fisher, Pittsburgh, PA), and coverslipped with toluene based mounting medium (Fisher, Pittsburgh, PA).

#### Immunostaining for Ki67 cell proliferation biomarker

Pancreatic tissue proliferation in response to injury was monitored by immunostaining of the nuclear proliferation marker Ki67. Pancreatic paraffin tissue sections were exposed to antigen-retrieval in boiling Tris-EDTA Buffer (10 mM Tris Base, 1 mM EDTA Solution, 0.05% Tween 20, pH 9.0), followed by antibody incubation and detection using the ABC-Vectastain system with the Vector DAB substrate kit (Vector Laboratories, INC, Burlingame, CA). Sections were counterstained with Eosin. Primary antibody was rabbit anti-Ki67 (Abcam, Cambridge, UK). Quantification was performed by counting Ki67 positive cells in an entire tissue section using a 20× objective. The image of the whole tissue section was captured with a digital camera (Canon, PowerShot, ELPH 300HS). The total area (mm^2^) of the entire tissue sections was measured with the Image J program (v1.46r; NIH), and the number of Ki67 positive cells per mm^2^ area was determined.

#### Pancreas Histological Severity Scoring (HSS)

Hematoxylin-eosin (H&E) staining was performed on 5 μm paraffin embedded pancreatic sections. Severity of pancreatic tissue damage was graded by a semi-quantitative scoring system (HSS). Within pancreatic sections, areas of abnormal pancreatic tissue architecture were graded: global glandular degeneration (acini and islets), vacuolization (fat deposition), acinar and islet cell atrophy including focal atrophy, area atrophy; and fibrosis: periductal, interlobular and intralobular fibrosis. These parameters were graded as follows: 0, absent; 1, minimal (<10%); 2, moderate (30 - 50%); and 3, major (>50 - 70%) percentage of the entire pancreatic section examined, as described by others previously [25–27, 45, 46]. Sections were observed and graded by two examiners blinded to animal treatment. A total score was calculated for both pancreatic head and tail.

#### Quantitative analysis of pancreas fibrosis with image J

The fibrous collagen in 5 μm-thick pancreas sections was stained with Sirius red. With this type of histological staining, the fibrous collagen deposition was stained red. Bright-field, polarized, TIFF images from the pancreas head and tail were acquired from consecutive non-overlapping fields using a Nikon E1000 microscope equipped with a Nikon DXM1200F digital camera and ACT-1 Program (Nikon Instruments, Inc., Melville, NY). Five images of each section were taken with 20× magnification at full resolution with a single image dimension setting of 3600 × 2880 pixels. The percentage of fibrotic tissue area was compared to the total area of tissue within an image. Mean values were obtained from 5 images of pancreas sections for each animal for comparison of groups.

Quantitative analysis of pancreatic fibrosis was performed using the Image J program (v1.46r; NIH) with the color thresholding plug-in. The threshold is set specifically for the Sirius red stained component (fibrosis). After setting the scale, the percentage of fibrosis was calculated by determining the tissue areas occupied by collagen fibers versus the total pancreas tissue areas, excluding the lumen (i.e., empty spaces) [47, 48] (Reinking L, 2007 *Image J Basics*http://rsbweb.nih.gov/ij/docs/pdfs/ImageJ.pdf; *Examples of Image Analysis Using ImageJ*http://rsb.info.nih.gov/ij/docs/pdfs/examples.pdf). The fibrosis % (of total tissue) = (area of fibrosis /area of total tissue) x100, i.e. = (area of entire image- non threshold area) / (area of entire image- area of empty space) × 100.

### Pain related behavioral assessments

Weekly behavioral testing was performed during the animal’s dark cycle active period (i.e. 0900 h – 1500 h). Investigators performing the behavioral studies were blinded to animal treatment.

#### Assessment of hindpaw secondary mechanical allodynia

Secondary mechanical allodynia on the hindpaws was assessed by measuring the mechanical withdrawal threshold using von Frey filaments with the “up - down” method described by Chaplan et al. [49]. Animals were placed on a raised wire mesh table (76 ×38 cm), under a clear plastic ventilated rat restrainer (18 ×13 ×15 cm) for a 30 min acclimatization period. The mechanical withdrawal threshold testing was done on the plantar surface of both hindpaws using a set 8 of von Frey monofilaments. The von Frey filaments were applied perpendicularly to the plantar surface with sufficient force to bend the monofilament slightly and held for about 5 seconds. A positive response was defined as an abrupt withdrawal (flick response) of the foot during stimulation or immediately after the removal of stimulus. Whenever there was a negative or positive response, the next stronger or weaker filament was applied, respectively. The pattern of positive and negative responses was converted into a 50% threshold value (in grams) using a curve-fitting algorithm [50]. The 50% decreased paw withdrawal threshold (PWT) indicated secondary mechanical allodynia.

#### Assessment of abdominal secondary heat hyperalgesia

Nocifensive responses to heat stimuli were tested by measuring the abdomen withdrawal latency with the modified Hargreaves test [51]. The radiant heat source was shone onto the abdominal skin of the reclining rats. Rats’ abdominal skin was shaved one day before the test each week. Rats were placed in separate clear plastic ventilated restrainers (18 ×13 ×15 cm) on a glass-top table (approximately 2 mm thick glass) and allowed to acclimate to their new environment for 30 min before testing. A high-intensity light beam was applied to the shaved abdominal skin surface through the glass and the latency of reflexive withdrawal responses timed (seconds). The cutoff time for abdominal withdrawal latency was set at 40 s to avoid any damage to the skin. A withdrawal event to radiant heat was defined as a weight shifting (either abdominal musculature contraction or lifting of the abdomen through postural adjustment) accompanied by head turning toward the stimuli and licking of the abdominal area. The abdomen withdrawal latency was tested in 3 trials for averages. The 5-min intervals between trials and a cooling fan under the table assured the temperature of the glass surface returned to room temperature (22 - 24°C) prior to the next trial. Shortened abdomen withdrawal latency values indicated a secondary heat hyperalgesia.

#### Modified 44°C hotplate test

Because C-fiber nociceptors are active under sustained, low-intensity heat stimulation, the modified hotplate test assessed responses to moderately noxious stimuli [52, 53]. In this study, two temperature controlled hotplates were topped with vented Plexiglas enclosures (26 cm × 26 cm × 28 cm) (Columbus Instruments, OH). One hotplate temperature was set at 38°C for pre-warming to normalize all animal paw temperatures, and the other one was set at 44°C. After a 30 min acclimation to the environment, rats were placed on the 38°C warm-up plate for 10 min. Then the rats were placed on the 44°C test plate for a 10 min test period. The latency to the first hindlimmb withdrawal response, the duration, and the frequency of hindlimb withdrawal events during heat stimulation were recorded. Event duration with hindpaws withdrawn from the plate began when the limb was lifted and finished when the limb made contact with the plate again. The events were plotted against time at 1 min intervals and a leftward shift of the event/time curve was an index of heat hyperalgesia. Erect leaning posture with forelegs against the wall of the enclosure (rearing event) was recorded. Rearing events were considered a noxious-evoked escape response during the hotplate test [54, 55].

#### Exploratory activity testing

Open field exploratory activity of the animals was monitored using the automated Flexfield Animal Activity System (San Diego Instruments, San Diego, CA) with Photobeam Activity System software (PAS) coupled to a HP computer (Hewlett Packard, Palo Alto, CA). The activity enclosure included a transparent Plexiglas chamber (40 × 40 ×40 cm) equipped with infrared photobeam sensors, 16 beams on each axis ( X and Y, total 32), arranged 4 cm above the chamber floor. Obstruction of these photobeams constitutes movements in the x and y planes. Another set of 16 beams is located 12 cm above the chamber floor to record movements in the z-plane (rearing events and duration). Data were collected for 45 minutes in nine 5-minute intervals.

The experiment was carried out in an isolated, temperature controlled (21 - 22°C) room, with consistent background white noise. When the tests started, the observer exits the room leaving the subjects undisturbed. Animals were tested at the same time of the day (from 9 am to 3 pm). To avoid acclimation of animals to the environment, repeat testing of the same animal occurred at least 24 h after the last test [56]. Six main parameters were measured in the nine 5 min intervals: rearing events and rearing duration; total activities, i.e., number of photobeams broken (included the number of different zone entries and stationary fine movement, e.g., grooming) and distance traveled; active time and resting time. Resting time is defined as a period when the animal remained in place for 1 second or longer. This test is based on the natural exploratory or investigatory behaviors of rodents in a novel “open field” environment. All of the six parameters are important for evaluation of spontaneous pain and for comparison of the effects of drug treatment. Changes in activity may not be reflected by a single parameter; therefore, each of the six parameters was evaluated in the comparisons [57, 58]. The test chambers were cleaned with Vindicator disinfectant cleaner (Hillyard, Inc) between tests to eliminate urine and other olfactory cues from previous subjects.

#### Light/dark preference test

A light/dark box with two equally divided compartments (26x13x28 cm) was used to assess anxiety-like behaviors of the rats [42, 59]. The two chambers were connected by a 10 × 10 cm doorway in the center of the partition to allow free access to the adjacent chamber. Rats were moved into the test room with the test apparatus 30 min prior to the test. All tests are conducted between 9 am and 11 am. Animals were placed initially in the dark chamber facing away from the door leading to the adjacent light chamber; and behaviors were recorded for 10 min (600 s). The following behavioral parameters were documented: the number of chamber crossings (light/dark chamber transition), defined as at least three of the animal’s paws stepping through the doorway from one chamber to the next; and total time (sec) spent in the light chamber.

### Statistical analysis

The data are expressed as means ±S.E.M. Comparisons among groups were performed with a One-way analysis of variance (ANOVA) followed by Tukey’s multiple comparisons post-tests. The time course comparison among groups was performed with a Two-way ANOVA with Bonferroni post-tests using GraphPad Prism version 6.0 (GraphPad Software, San Diego California USA). Two-tailed t-tests were also used where appropriate. A *p* ≤ 0.05 was considered significant. Calculation of the correlation coefficient between tissue HSS and pain related behaviors was done with the Microsoft Excel program.

## Abbreviations

ABWL: Abdominal withdrawal latency
AHF: Alcohol/High fat diet
ANOVA: Analysis of variance
CB2: Cannabinoid receptor 2
GABA: Gamma amino butyric acid
HSS: Histological severity scoring
PWT: Paw withdrawal threshold.
